# Supplementary figures and images for: Alternating hemiplegia of childhood: An electroclinical study of sleep and hemiplegia
Source: PLoS One. 2022 Sep 30;17(9):e0268720. doi: 10.1371/journal.pone.0268720 (PMC9524638; doi:10.1371/journal.pone.0268720)

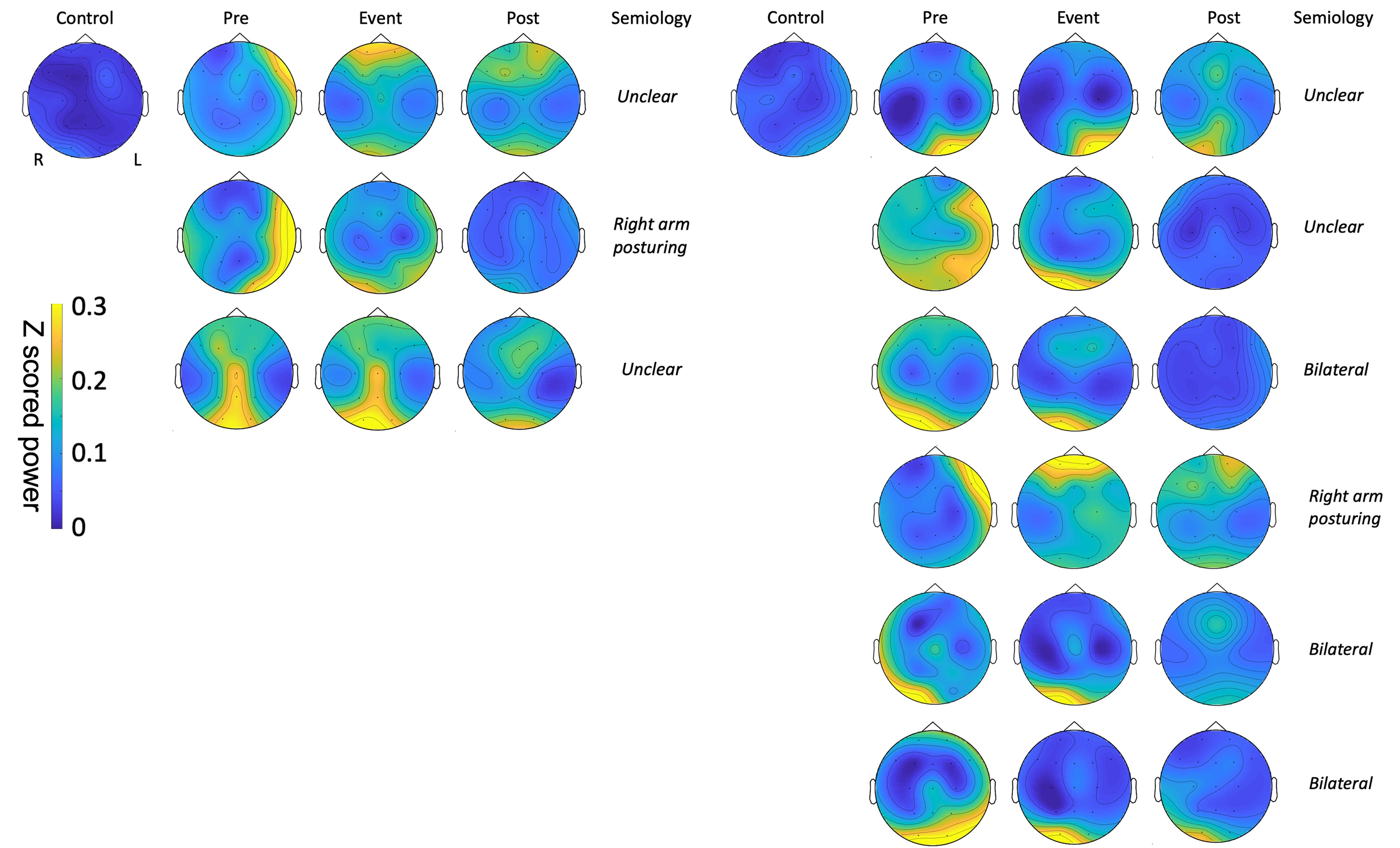

Supplement: S1 Fig — Topographic distribution of normalised EEG power between 1 and 10 Hz at baseline, pre-event (5 minutes immediately preceding clinical onset), event (5 minutes during hemiplegic episode) and post-event (5 minutes immediately following clinical offset) epochs from all hemiplegic episodes analysed. From top to bottom: Patient 3 events 1–3 (left) and Patient 2 events 1–6 (right) and. Beside each event is a clinical lateralisation of attack (semiology). The colour bar represents differences in normalised power (arbitrary units), with increased power represented by yellow colours and decreased power represented by blue powers. R = Right; L = Left. (TIF) [file pone.0268720.s001.tif]
