# Supplementary material for: Alternating hemiplegia of childhood: An electroclinical study of sleep and hemiplegia
Source: PLoS One. 2022 Sep 30;17(9):e0268720. doi: 10.1371/journal.pone.0268720 (PMC9524638; doi:10.1371/journal.pone.0268720)
Supplement: S1 Table — (DOCX) [file pone.0268720.s002.docx]

| **Control ID** | **Age (years)** | **Gender** | **Treatment at the time of EEG** |
| --- | --- | --- | --- |
| 1 | 33 | Female | Not on medications |
| 2 | 20 | Female | Levetiracetam, propranolol, mirtazapine, loratadine |
| 3 | 20 | Female | Clobazam, clonazepam, lamotrigine, lacosamide, topiramate, promethazine, procyclidine |
| 4 | 24 | Female | Valproate, clobazam, phenytoin, carbamazepine, quetiapine |
| 5 | 23 | Female | Clobazam |
| 6 | 39 | Female | Valproate, levetiracetam |
| 7 | 49 | Female | Topiramate, lacosamide |
| 8 | 19 | Female | Fluoxetine |
| 9 | 23 | Female | Levetiracetam, valproate, propranolol |
| 10 | 30 | Female | Topiramate, lamotrigine, propranolol, loratadine, omeprazole |
| 11 | 17 | Female | Levetiracetam, clobazam, pyridoxine, omeprazole |
| 12 | 34 | Female | Levetiracetam, lamotrigine, valproate, metoclopramide |

**Supporting Table 1. Age, gender and treatment at the time of EEG of the epilepsy control cohort.**
