# Supplementary material for: Alternating hemiplegia of childhood: An electroclinical study of sleep and hemiplegia
Source: PLoS One. 2022 Sep 30;17(9):e0268720. doi: 10.1371/journal.pone.0268720 (PMC9524638; doi:10.1371/journal.pone.0268720)
Supplement: Attachment — Submitted filename: Response to Reviewers PLOSONE.docx [file pone.0268720.s004.docx]

PONE-D-22-12955

ALTERNATING HEMIPLEGIA OF CHILDHOOD: AN ELECTROCLINICAL STUDY OF SLEEP AND HEMIPLEGIA

Dear Editors,

Thank you for sending our manuscript for peer review, and for giving us the opportunity to address the reviewers’ comments. Below we provide detailed answers to the comments made by the reviewers, and tracked all the changes in the manuscript. In addition, we addressed the journal requirements as requested.

Reviewer #1: We are grateful that the reviewer found the data clearly reported, statistically analyzed and appropriately discussed.

*Both AHC patients and epilepsy controls were taking drugs at the time of the study. This represents an insurmountable limit of the paper. I think that the Authors should specify in the text or in a supplementary file which drugs the epilepsy controls were taking. Also in the Table 1 another column could contain the drugs taken by the AHC patients.*

We thank the reviewer for this important point. We have now added treatment data for both patients and epilepsy controls in Table 1 and Supporting Table 1, respectively. We also wrote in the discussion (page 9):

‘Another potential source of confounding was the complex polytherapy, mostly comprising antiseizure medications, that both the patients with AHC and the epilepsy control group received. This could have affected the EEG power and arousal data.’

Reviewer #2: We agree with the reviewer that AHC is a fascinating disorder where seizures are frequent but ill-understood other paroxysmal phenomena occur, most prominently hemiplegia, that resolves with sleep.

*While sleep has been studied in this disorder, the novel aspect of this paper is the examination of adult subjects and the more detailed spectral analysis performed on hemiplegic attacks, although only 2 of the 5 cases had these. The clinical features of the 5 cases are typical of AHC. The description of the telemetry recordings of the events is useful although this was done retrospectively and eye movement monitors were not used preventing scoring of REM sleep.*

*Key findings were lateralization on EEG during the events where this was interpretable and spectral clots documenting the hemispheric asymmetry during events. A change in spectral power between AHC and control groups was found in the awake state only, the significance of this is a little unclear.*

*It would be useful if the lateralization could be more clearly described. For example, in table 3, the hemispheres are divided up in to those with “increased activity” and “reduced activity” but it is not immediately clear whether which hemisphere is relevant to the hemiplegic limb. Similarly, with the topographic maps shown on figure 1, it is not really clear what side of the body was paralysed in relationship to the evolution of the spectral power pre and post event and this should be clarified.*

We thank the reviewer for this important distinction. Even with experienced neurophysiologists reviewing the attacks, due to the complex nature of them, it was challenging to definitively assign clinical lateralisation to the majority. Indeed normal hemi-function was demonstrated in two events where we can be certain about lateralisation. In these two cases the corresponding lateralisation of spectral power (i.e. contralateral to clinical semiology) was demonstrated. What is interesting is that nearly all attacks demonstrated prior spectral lateralisation irrespective of clinical semiology, which argues the possibility that attack onset has a focus. For this reason, we used increased/reduced spectral activity as a comparator in order to permit statistical analysis. For ease of understanding we have outlined each of the attacks in Table 2, and amended Figure 1 and Supplemental Figure 1 to incorporate clinical semiology when possible.

*Under ‘significance’ the authors state that disrupted sleep is related to SUDEP. It is quite unclear what the data in this paper adds to the understanding of SUDEP, in general or in AHC and it is nor even properly discussed. I recommend this be deleted.*

We have now removed the statement on SUDEP, as suggested.
